# Global potential distribution prediction of *Xanthium italicum* based on Maxent model

**DOI:** 10.1038/s41598-021-96041-z

**Published:** 2021-08-16

**Authors:** Yang Zhang, Jieshi Tang, Gang Ren, Kaixin Zhao, Xianfang Wang

**Affiliations:** 1 College of Computer Science and Technology, Henan Institute of Technology, Henan, 453003 China; 2 Institute of Data Mining and Intelligent Computing, Henan Institute of Technology, Henan, 453003 China; 3 College of Life Science, Sichuan University, Chengdu, 610000 China

**Keywords:** Biological techniques, Environmental sciences

## Abstract

Alien invasive plants pose a threat to global biodiversity and the cost of control continues to rise. Early detection and prediction of potential risk areas are essential to minimize ecological and socio-economic costs. In this study, the Maxent model was used to predict current and future climatic conditions to estimate the potential global distribution of the invasive plant *Xanthium italicum*. The model consists of 366 occurrence records (10 repeats, 75% for calibration and 25% for verification) and 10 climate prediction variables. According to the model forecast, the distribution of *X. italicum* was expected to shrink in future climate scenarios with human intervention, which may be mainly caused by the rise in global average annual temperature. The ROC curve showed that the AUC values of the training set and the test set are 0.965 and 0.906, respectively, indicating that the prediction result of this model was excellent. The contribution rates of annual mean temperature, monthly mean diurnal temperature range, standard deviation of temperature seasonal change and annual average precipitation to the geographical distribution of *X. italicum* were 65.3%, 11.2%, 9.0%, and 7.7%, respectively, and the total contribution rate was 93.2%. These four variables are the dominant environmental factors affecting the potential distribution of *X. italicum*, and the influence of temperature is greater than that of precipitation. Through our study on the potential distribution prediction of *X. italicum* under the future climatic conditions, it has contribution for all countries to strengthen its monitoring, prevention and control, including early warning.

## Introduction

With the rapid development of economic globalization, international trade exchanges and human activities are becoming more and more frequent, alien invasive species are expanding at an unprecedented rate^1–3^. The resulting ecological problems and increasing risks of biological invasion have caused huge eco-environmental hazards and economic losses all over the world^4,5^. The biological invasion has become a great challenge for human beings. *Xanthium italicum* Moretti is one of the most widely distributed invasive plants in the world, and its biological characteristics of easily carrying seeds make it more likely to spread widely^6^. *X. italicum* is an annual herbaceous plant of the genus *Xanthium* in the Compositae family, propagated by seeds^7^, and native to North America but has been spread to many countries and regions in Europe, Asia, and Oceania^8,9^. Previous studies shown that *X. italicum* seeds have been found in old crawler excavators imported from Japan, rapeseed seeds from Mongolia, and soybeans imported from Brazil, Argentina and the United States^10–12^. Because of its strong ecological adaptability, *X. italicum* can grow in wasteland, fields, river beaches and ditch roadsides, and plants grow more luxuriantly and taller in wetland, irrigated land and ditches. *X. italicum* has high plant coverage and strong competitiveness, it can compete water, nutrition, light and growth space with native species, to form a dominant population in the habitats^13^. Moreover, *X. italicum* also has strong communicative power, and the involucre bears dense barbs, which can easily be attached to livestock and poultry, wild animals, agricultural machinery, seeds and packaging of agricultural by-products for long-distance transmission^9^. It caused serious harm to agriculture, animal husbandry and biodiversity in the invaded areas^14^. It has been reported that the *X. italicum* caused 43% and 90% of the yield loss after introducing sunflower and corn crops to agricultural production areas, respectively^15,16^. In some farming and pastoral areas, the large and prickly fruits of *X. italicum* are often carried by the fur of some livestock, which not only caused the further spread of *X. italicum*, but also reduced the quality of products such as wool for sale^17^. *X. italicum* has strong phenotypic plasticity to different environmental conditions, showing great invasive potential^18^. A clear understanding of the potential distribution area of *X. italicum* under global natural conditions has great significance for its monitoring, prevention and control, and early warning.

The niche model uses the well-known distribution data of species and relevant environmental variables, constructs the model in accordance with a deep learning algorithm, induces or simulates the ecological needs of species, and projects the calculation results at different time and space to predict the potential distribution of species^19,20^. At present, the niche models to predict species potential distribution areas mainly include the Genetic Algorithm for the Rule Set Production (GARP), Maximum Entropy (MAXENT), Match Climates Regional Algorithm (CLIMEX), Climatic Envelope (BIOCLIM)^21–24^. Each model has a different theoretical basis, data requirements and analysis methods. Most studies show that, compared with other niche models, the Maxent model not only has good prediction effect and stability but also has the advantages of simple and fast operation, and small sample demand, so it has become an ideal prediction tool for many scholars^25–28^. It builds a prediction model based on the actual distribution points and environmental variables of the distribution area stored in GIS, and to simulate the potential distribution of species in the target space. The output is a thematic map reflecting the suitability of the relative distribution of species^29,30^. Maxent model has been widely used in the field of species distribution research^31,32^, such as the potential distribution of *Haloxylon persicum* in Central Asia under global warming^33^, the prediction of the suitable growth zone of *Rhinopithecus roxellana* due to the sharp reduction of human disturbance^34^, and the distribution of five economic tree species in the Amazon River basin combined with remote sensing technology^35^. Besides, Maxent niche models have also been used to study the potential distribution of invasive species in recent years, such as *Solidago canadensis, Ageratina adenophora, Mimosa pigra, Flaveria bidentis, Solenopsis invicta* and *Ambrosia artemisiifolia*^36–41^.

As the prevention and control of *X. italicum* is an extremely time-consuming and costly project, the prediction and simulation of the potential distribution area of *X. italicum* is highly important for the future prevention and control management. In this paper, the distribution record points of *X. italicum* were determined by consulting the literature and GBIF website. In combination with the relevant environmental variables of *X. italicum* invaded area, taking the SSP245 scenario with human intervention as forecasting background, the global potential distribution area of *X. italicum* in current and future was predicted with the help of Maxent model tools to provide theoretical support for decision-makers to formulate corresponding prevention and control management measures.

## Materials and methods

### Distribution data

The foreign distribution data of *X. italicum* were downloaded from the Global Biodiversity Information Facility (GBIF) http://data.gbif.org/welcome.htm, while the data of China were mainly collected from literature and publications^7,13^. We queried the specific coordinates of the place in Geonames website (http://www.geonames.org/), deleted the duplicate and invalid distribution points, and finally got 336 effective distribution points. Detail coordinate point information was included in the supplementary material Table S1, and the species names and longitude and latitude of *X. italicum* distribution data were recorded in the Excel file as .csv format, in which the east longitude and north latitude were marked as positive values, and the west longitude and south latitude were marked as negative values.

### Selection and treatment of environmental variables

The sixth IPCC assessment report publishes four climate change scenarios, namely, SSP126 scenario, SSP245 scenario, SSP370 scenario, and SSP585 scenario. We chose the SSP245 scenario, where greenhouse gas emissions are about the same as current condition (1970–2000) and the global average temperature tends to reduce with human intervention. 19 environmental-climate factors derived from the WorldClim environmental database (http://www.worldclim.org/), with a spatial resolution of 5 km (Table 1).

**Table 1:** Environmental variables extracted from current *X. italicum* habitats.

Code	Variables description	Code	Variables description
Bio1	Annual mean temperature	Bio11	Mean temperature of coldest quarter
Bio2	Mean diurnal range	Bio12	Annual precipitation
Bio3	Isothermality	Bio13	Precipitation of wettest month
Bio4	Temperature seasonality	Bio14	Precipitation of driest month
Bio5	Max temperature of warmest month	Bio15	Precipitation seasonality
Bio6	Min temperature of coldest month	Bio16	Precipitation of wettest quarter
Bio7	Temperature annual range	Bio17	Precipitation of driest quarter
Bio8	Mean temperature of wettest quarter	Bio18	Precipitation of warmest quarter
Bio9	Mean temperature of driest quarter	Bio19	Precipitation of coldest quarter
Bio10	Mean temperature of warmest quarter		

**Figure 1:**
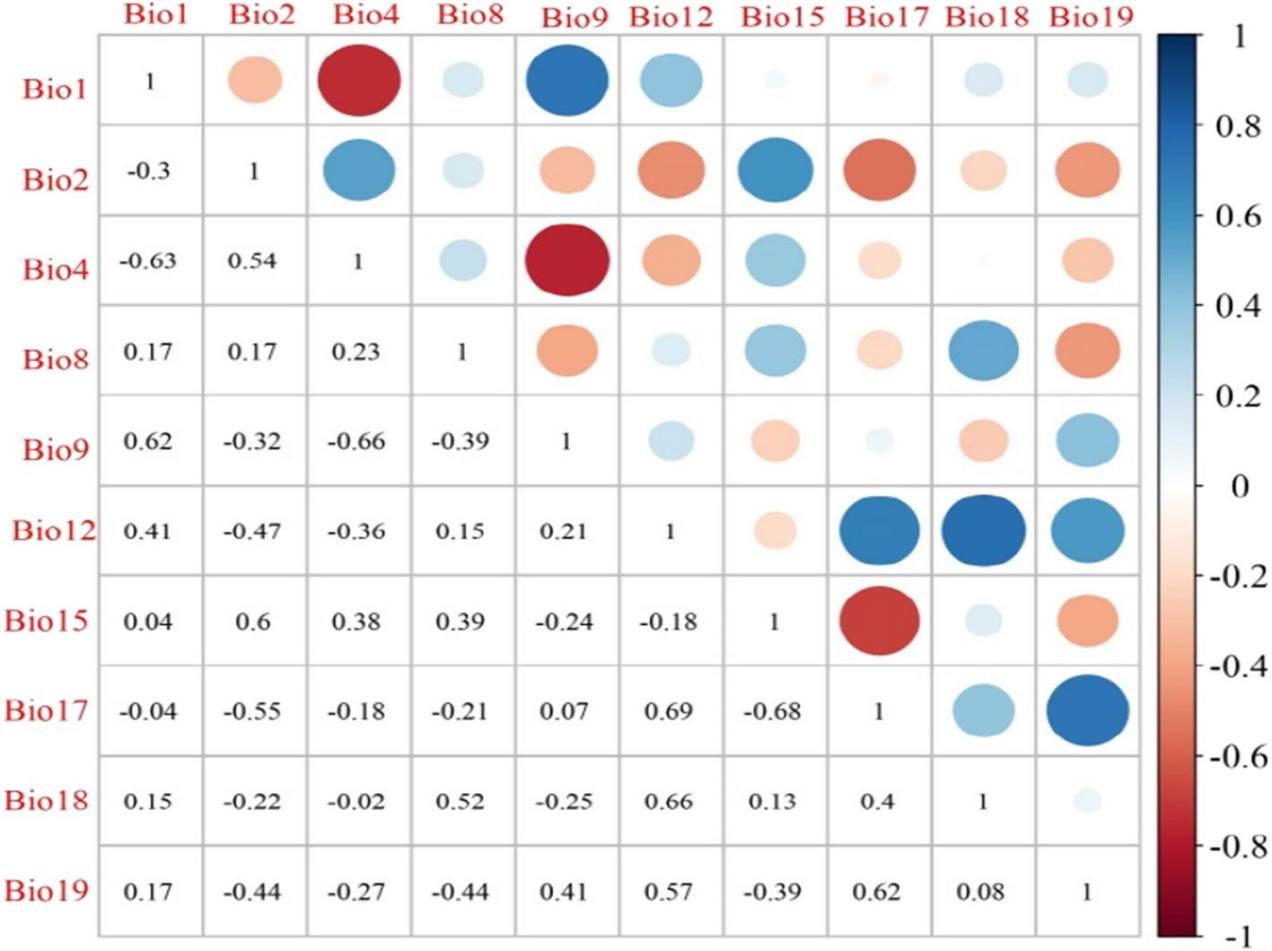
Pearson correlation analysis of various environment variables (r < 0.8). Created in R 4.0.2 (https://www.r-project.org/).

Environmental variable data is an important parameter for constructing a niche model, and using too many environmental variables to construct the model will enhance the spatial correlation between variables, cause over-fitting, and then reduce the transferability of the model. On the contrary, choosing the moderate and reasonable environmental variables can significantly improve the prediction ability of the model^42,43^. Therefore, it is necessary to calculate the correlation of environmental variables and exclude the high correlation environmental variables.Therefore, we used Pearson correlation analysis of R v4.0.2 software^44^ to screen 10 environmental variables whose correlation is less than 0.8 (Fig. 1, Table 2) and set them as environmental parameters into Maxent software^45^.

**Table 2:** Retained environmental variables used in this study.

Code	Variables description
Bio1	Annual mean temperature
Bio2	Mean diurnal range
Bio4	Temperature seasonality
Bio8	Mean temperature of wettest quarter
Bio9	Mean temperature of driest quarter
Bio12	Annual precipitation
Bio15	Precipitation seasonality
Bio17	Precipitation of driest quarter
Bio18	Precipitation of warmest quarter
Bio19	Precipitation of coldest quarter

### Map data and software sources

The global vector map comes from the website of the National Fundamental Geographic Information System (http://nfgis.nsdi.gov.en); the Maxent software version 3.3.3 k (http://www.Cs.princeton.edu/-schapire/maxent/); ArcGIS spatial technology platform is a complete set of GIS products developed by ESRI company in the United States, and the ArcGIS software version is 10.2.2^46^. Species current distribution was again overlaid on the Köppen–Geiger climatic classification system (1976–2000)^47^.

### Data processing

Import distribution data and environment data into Maxent v3.3.3, randomly select 25% of the distribution points as testing data, the remaining 75% as training data, and the model was trained for 10 repetition^48^. In the environment parameter settings, we used the Jackknife method, and other parameter settings followed software default values. The LHQPT feature classes were selected in this model and the regularization multiplier was set to 1.0^49^. The ENMeval package was used to calculate the corrected Akaike information criterion correction value (AICc value) under these parameters, and the “checkerboard2” method was used to calculate the AICc value^50^. The output was a grid layer of ASCII format, and the value of each grid in the layer represented the adaptation of *X. italicum* to the environment in that area, with a range of 0 to 1. We loaded the calculation result of Maxent in ArcGIS10.2.2 software for visual expression^51^.

### Model accuracy test

The receiver operating characteristic curve (ROC) analysis method was used to test the accuracy of this model. The ROC curve takes the true positive rate as the ordinate (the ratio that exists and is predicted to exist) and the false positive rate (the ratio that does not exist but is predicted to exist) as the abscissa. The AUC value is the area enclosed by the abscissa and ROC curve, and the range is 0–1. The larger AUC value is, the farther the distance from the random distribution is, the greater correlation between environmental variables and predicted geographical distribution of species is, and the better prediction effect of this model is. On the contrary, it means that the prediction effect of the model is worse. The AUC value of 0.5–0.6 means the simulation effect of this model is failed; 0.6–0.7 means the simulation effect is poor; 0.7–0.8 means the simulation effect is average; 0.8–0.9 means the simulation effect is good; 0.9–1 means the simulation effect is perfect^52,53^. Moreover, three other approaches also have performed in the supplementary table S2 to test the performance of Maxent model, which includes kappa coefficient (K), Normalized Mutual Information (NMI) n(s) and True Skill Statistic (TSS)^30,54,55^.

### Ethics approval

This paper evaluates published data and does not need specific ethics approvals.

### Consent to participate

Human subjects were not involved in this study.

### Consent for publication

Human subjects were not involved in this study.

## Results

### Global potential distribution prediction of *X. italicum*

**Figure 2:**
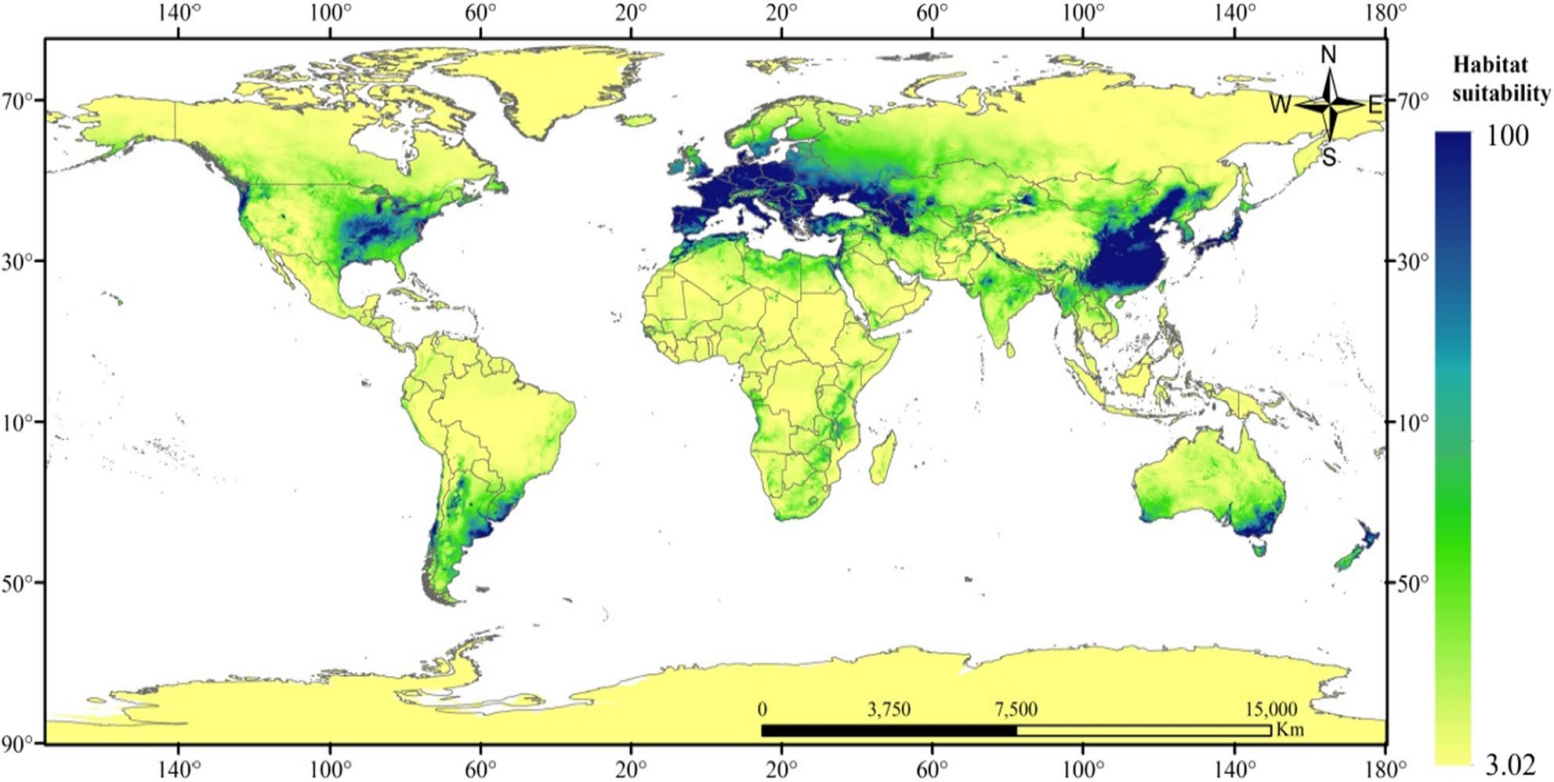
Current potential binary distribution of *X. italicum* under normal climate (1970–2000) according to the simulations. Created in ESRI ArcMap 10.2.2 (https://support.esri.com/en/Products/Desktop/arcgis-desktop/arcmap/10-2-2#downloads).

**Figure 3:**
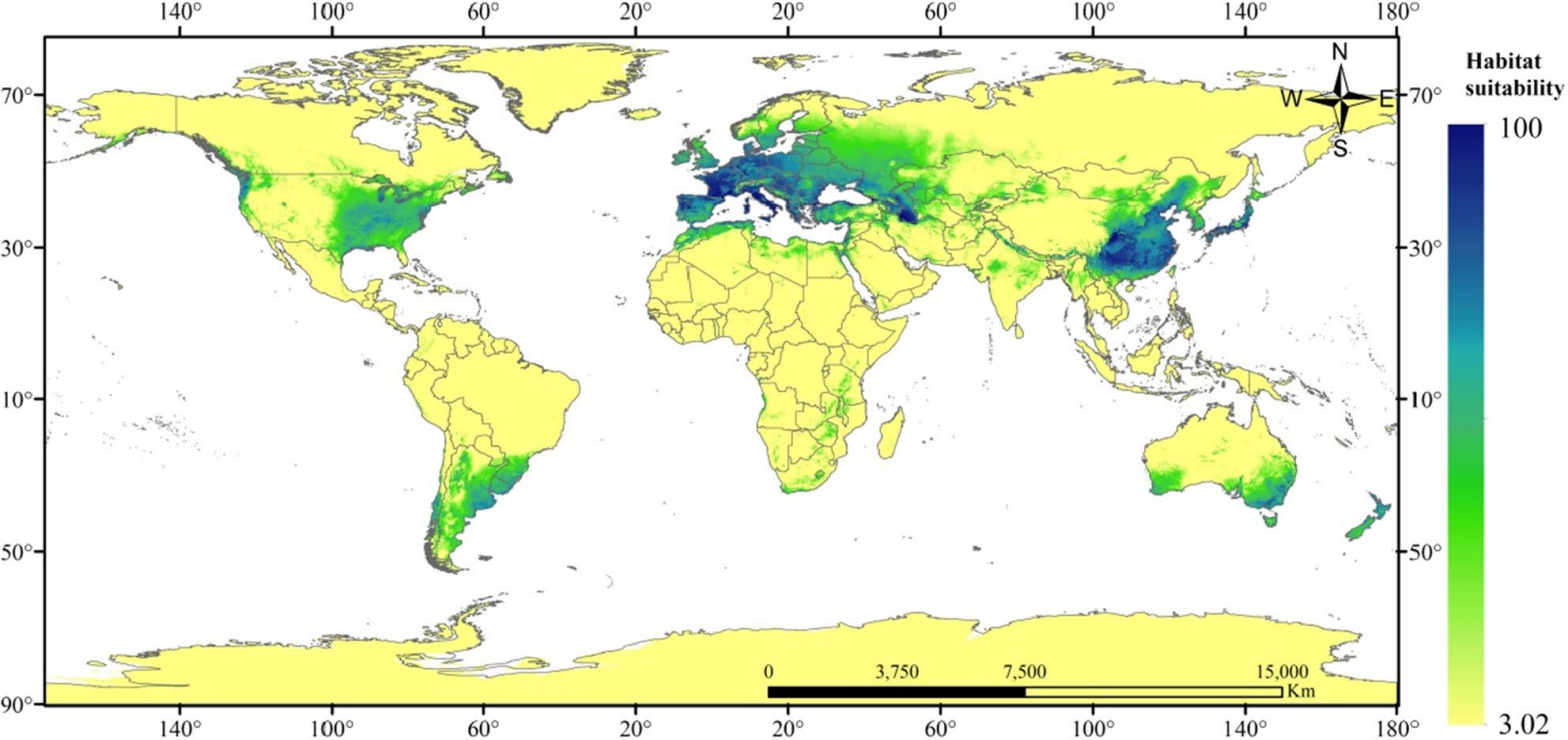
Future potential binary distribution of *X. italicum* under SSP245 scenarios (2050) according to the simulations. Created in ESRI ArcMap 10.2.2 (https://support.esri.com/en/Products/Desktop/arcgis-desktop/arcmap/10-2-2#downloads).

**Figure 4:**
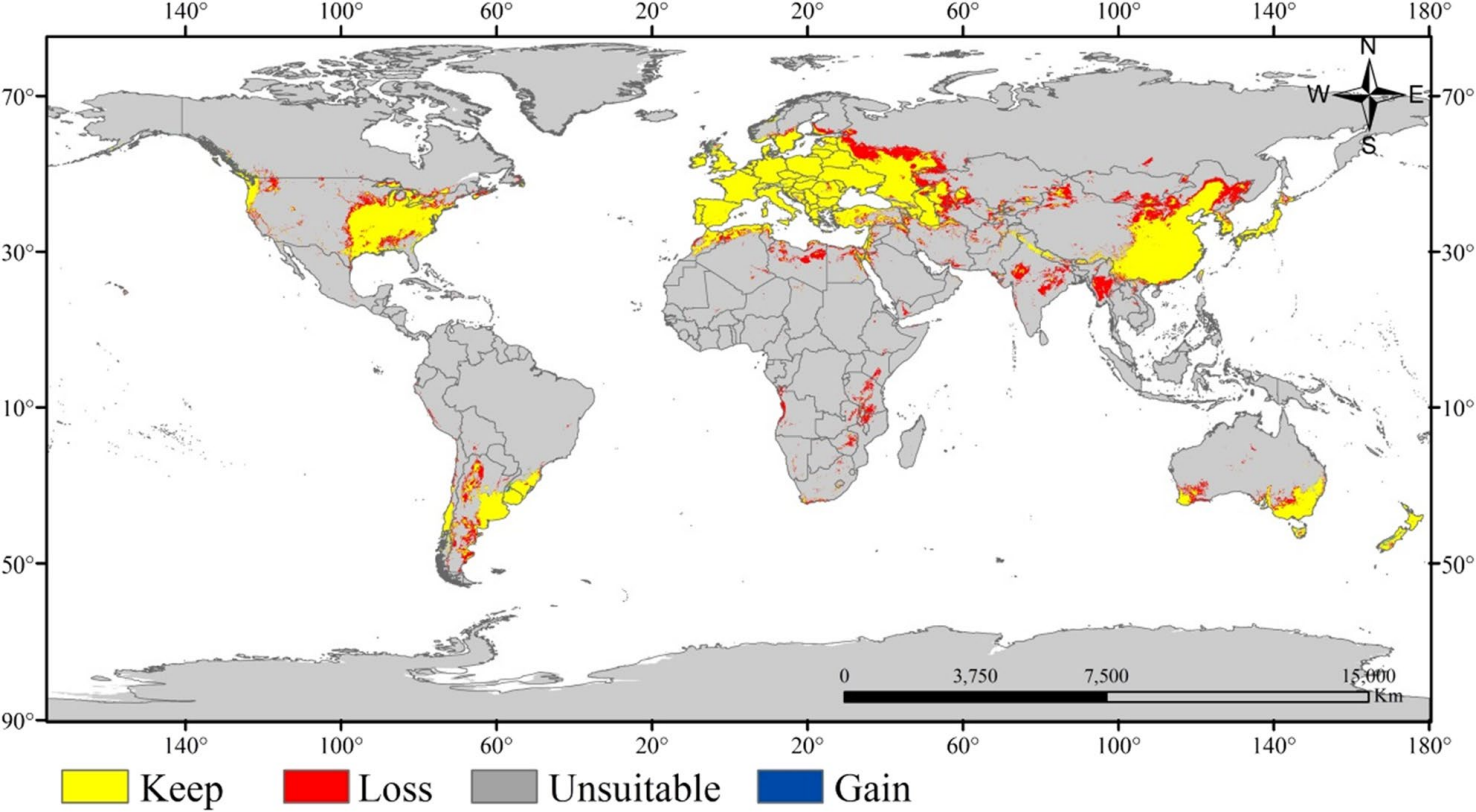
Changes in the potential habitat suitability of *X. italicum.* Created in ESRI ArcMap 10.2.2 (https://support.esri.com/en/Products/Desktop/arcgis-desktop/arcmap/10-2-2#downloads).

In the base climate (1970–2000), the suitable growth area prediction result of *X. italicum* was shown in Fig. 2. In the SSP245 scenario (2050), the potential distribution prediction of *X. italicum* was shown in Figs. 3 and 4 showed the change of suitable growth area between these two maps. As it can be seen from Fig. 4, the global suitable area of *X. italicum* in the future (17.8650 million km^2^) is less than that the present (19.4315 million km^2^), which relatively reduces the pressure on the ecological environment in some areas, but its invasion potential cannot be ignored. Therefore, it is extremely unwise to relax the early warning of *X. italicum*. Except for Antarctica, there are suitable zones for *X. italicum* on all continents.

The suitable growth area of *X. italicum* is mostly concentrated in the north–south temperate zone, rarely distributed in the tropics and no distribution in frigid zones. From the potential distribution range of *X. italicum*, it prefers temperate marine climate, Mediterranean climate, and temperate continental monsoon climate, while the distribution area in temperate continental climate is obviously reduced. So moisture may be a key factor affecting the potential distribution of *X. italicum*.

### Accuracy evaluation of Maxent model

**Figure 5:**
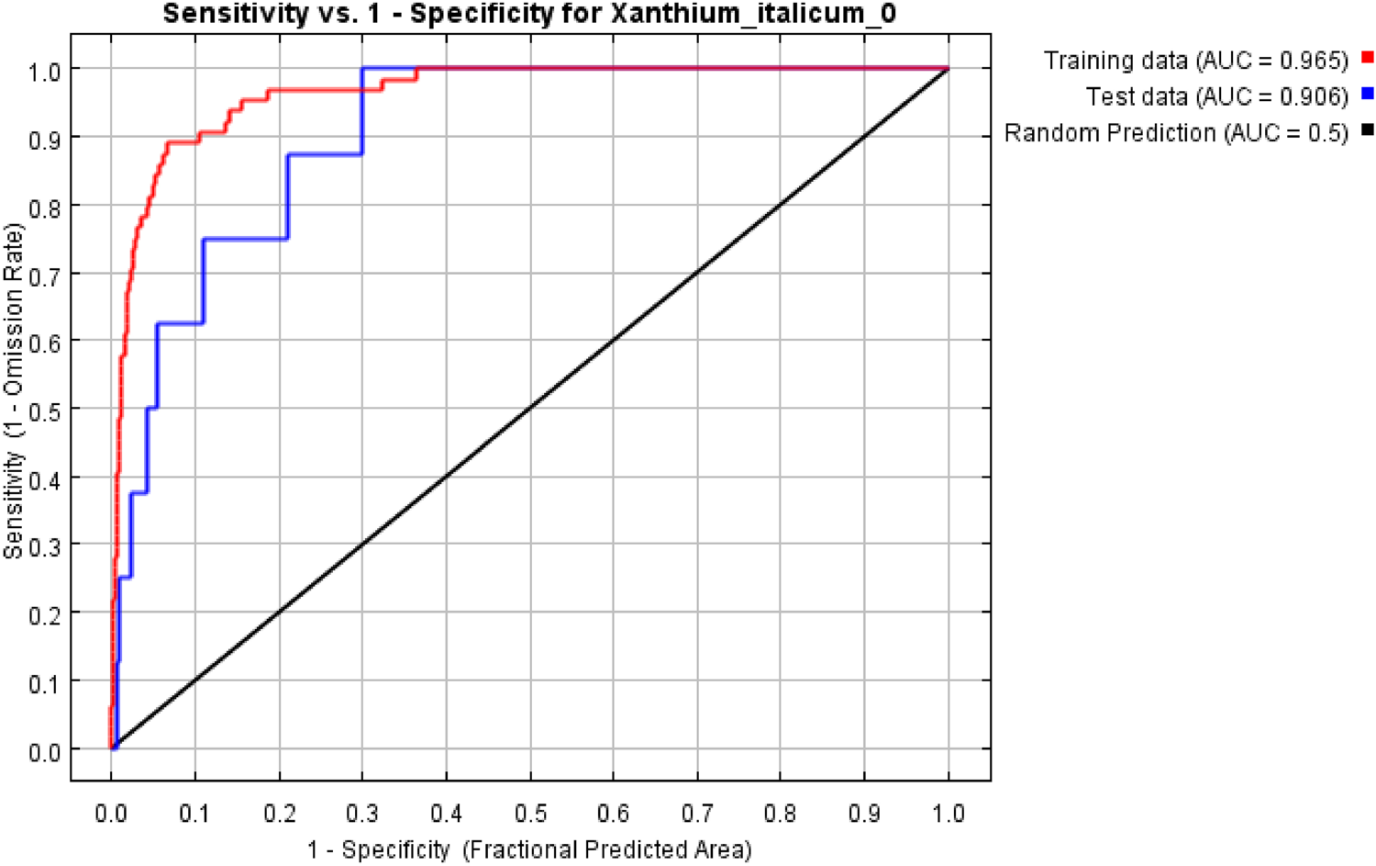
Receiver operating characteristic (ROC) curve and AUC values of the MaxEnt model. The red curve indicates training data, the blue curve indicates test data, and the black line indicates random prediction.

The final ROC curve of this model was shown in Fig. 5. In this study, the AUC values of training data and verification data are 0.965 and 0.906 respectively, indicating that the Maxent model is very effective in predicting the global potential distribution of *X. italicum*. The predicted geographical distribution results and the actual distribution have a high degree of overlap between the regions of *X. italicum*, which means the results can be applied to the suitable regionalization of this species.

### Weight analysis of dominant environmental variables

**Figure 6:**
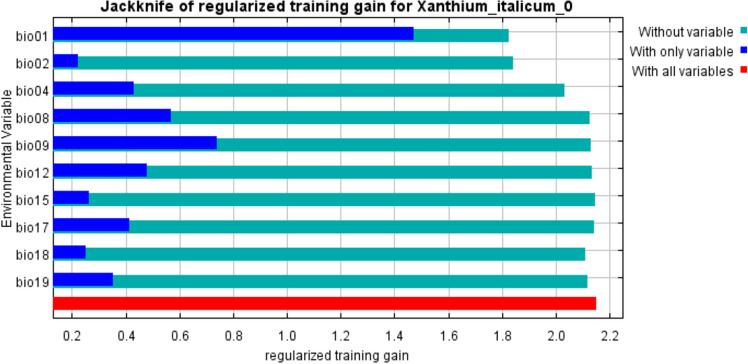
Important analysis of environmental variables based on Jackknife tests.

Jackknife method was used in the Maxent model, and the results can show the weight of different environmental factors affecting the habitat suitability of *X. italicum* (Fig. 6). The vertical axis represents the screened environmental variables, and the horizontal axis represents the score of each environmental variable. The dark blue column represents the model score with only this environmental factor exists, and the light blue column represents the sum of the scores of other variables except for this variable, and red represents the sum of all variables scores. As can be seen from Table 3, the main environmental factors affecting the potential distribution of *X. italicum* are annual mean temperature, monthly mean diurnal temperature range, the standard deviation of temperature seasonal change and annual average precipitation. The contribution rates are 65.3%, 11.2%, 9.0% and 7.7% respectively, and the total contribution rate is 93.2%. The annual mean temperature has the highest contribution rate, which indicates that the annual mean temperature is the most important factor affecting the potential distribution of *X. italicum*.

**Table 3:** The percentage contribution of ten climatic variables for *X. italicum* based on Jackknife tests.

Variable	Contribution (%)
(Bio1) Annual mean temperature	65.3
(Bio2) Mean diurnal range	11.2
(Bio4) Temperature seasonality	9.0
(Bio12) Annual precipitation	7.7
(Bio19) Precipitation of coldest quarter	2.7
(Bio9) Mean temperature of driest quarter	1.7
(Bio18) Precipitation of warmest quarter	1.5
(Bio17) Precipitation of driest quarter	0.5
(Bio8) Mean temperature of wettest quarter	0.4
(Bio15) Precipitation seasonality	0

## Discussion

The Global Invasive Species Program (GISP) has proposed that preventing the invasion of alien species is more feasible and economical than controlling its outbreak^56^. Therefore, it is quite importance to understand the climate-driven changes on the potential distribution and range shifts of this plant for better planning and decision-making of control and management actions. Using all available global occurrence data and reliable species responsive environmental predictors, we demonstrated that the overall potential suitability for *X. italicum* will contract in the future under SSP245 scenarios relative to the current climate (Fig. 4). The simulation results showed that the suitable growth range of *X. italicum* is very wide in the world, and it is mostly concentrated in the north–south temperate zone. Although the potentially suitable zone of *X. italicum* in 2050 is less than the current predicted distribution range, it still has great potential for invasion. Some countries, such as China, South Korea, Japan, the United States, Australia, Angola, Namibia, the Republic of Mozambique, Kazakhstan and European countries all have high suitable zones of *X. italicum*, which has a very high risk of invasion. This was consistent with the information that has been reported and various countries government should attach great importance to this species^7,57^.

Distribution and modelling results can also be influenced by other intrinsic factor (dispersal distance and rate of the species, and its generation time) and extrinsic factor (human activity and natural enemy)^50^. According to the analysis of the variable contribution and the Jackknife test (Table 3; Fig. 6), the temperature and precipitation were found to be a significantly important variable in determining the distribution of this plant. Climate, particularly temperature, is the main factor that controls the distribution of biological invaders on earth^58^. Among bioclimatic variables, the main environmental factors that have a great influence on the distribution of *X. italicum* are annual mean temperature (65.3%), monthly mean diurnal temperature range (11.2%), the standard deviation of temperature seasonal change (9.0%) and annual average precipitation (7.7%). The first three were all environmental factors related to temperature, and the contribution percentage of annual average precipitation is only the fourth, which showed that the contribution rate of temperature factor to the geographical distribution of *X. italicum* was greater than that of precipitation factor. This was consistent with the results of literature reports and field investigation, *X. italicum* can grow in wetlands, farmland, deserts and other environments with great differences in soil moisture, its relative anti-waterlogging, and drought-resistant growth characteristics lead to the contribution rate^7,9,18^. Whether temperature can be used to slow down or stop the trend of rapid invasion of *X. italicum* remains to be verified by further research.

Based on the SSP245 scenario, we predicted the global suitable growth region of *X. italicum* in 2050. Among these four climate scenarios, the SSP245 scenario is the closest scenario to reality. The predicted reduction of potential geographical distribution may be due to the increase of global annual mean temperature with human intervention because the annual mean temperature accounts for a very high percentage of all ecological factors, and the temperature factor has a significant effect on the growth of *X. italicum*. Although the global total suitable area of *X. italicum* has decreased, the suitable area of temperate maritime climate, Mediterranean climate, and temperate continental monsoon climate have not increased. It indicated that the contribution percentage of annual average precipitation to *X. italicum* distribution is not high, but it still has a certain impact on *X. italicum* growth. A possible explanation is that the regions with a temperate maritime climate, Mediterranean climate, and temperate continental monsoon climate are relatively humid, and the temperature of humid air is more stable than that of dry air^59^. Therefore, the annual mean temperature does not decrease significantly in the above-mentioned areas, so did not cause a change in the suitable area of *X. italicum*. Our habitat suitability maps indicate a highly conducive environment for *X. italicum*, these areas are predicted to be found rather steady under future climate scenarios as well (Fig. 5).

In recent years, ROC curve analysis has been widely used in the evaluation of species potential distribution prediction models, especially in invasive species^60–63^. In this paper, the performance of the MaxEnt model was revealed by the ROC curve, whereas the accuracy of the prediction mapping (current and future) was determined by the percentage contribution and jackknife test. Additionally, classification accuracy measures, such as K, NMI and TSS, were in agreement with Bhandari^30^. Further, it concurs about the sample size of the studied species to determine the training and testing of geo-coordinates used for prediction mapping (Supplementary material Table S2). Thus, Our results showed that the simulation effect of this model is very good, and can accurately simulate the global distribution of *X. italicum*. However, the premise of applying a niche model is to assume that species niche demand is conservative, but the niche drift of invasive species sometimes occurs, there may produce a deviation in the potential distribution of *X. italicum* after niche drift^64,65^. Moreover, because alien species usually spread from one or several locations of the earliest invasion, in a specific time, the ecological characteristics of alien invasive species in the introduction area are difficult to reflect the complete ecological needs of species. Using the non-equilibrium distribution data of the intrusion site, especially the early distribution data, there will be an error in the prediction of suitable growth areas^4,66^. It may be more accurate to use the equilibrium distribution data of invasive regions to predict other suitable growth areas. So, the demography of this species requires us to follow up the invasion area, but it may not be allowed in some countries. At present, many countries pay less attention to *X. italicum*, and the report of this species is not detailed enough. Therefore, it is important to strengthen the quarantine control on the importation of commodities, especially of transport vehicles and goods at potential donor spots (i.e., border control/border biosecurity system), to decrease further risks of this biological invader.

Invasive plants respond to the changing climate i.e., increases in temperatures and CO_2_ levels, changes in precipitation^67^. Thus, long-term changes in the climate can have potential influences on the distribution of *X. italicum* habitats due to the limitation of suitable climate conditions. At present, this plant need immediate and ongoing control and management measures as the current predicted potential ranges are very high. This study provides insights for decision-makers that climate change influences on potential distribution of invasive species should be considered for long-term effective management of this species. Our results also provide detail information relevant to potentially suitable areas of this invasive plant in current and future under climate change. Defining likely spread areas and recognizing the pattern of invasion in the future are important components of climate change-integrated short-term and long-term conservation management strategies. The study has mapped the areas potentially suitable for the distribution of *X. italicum* across the worldwide under current and future climate scenarios. Administrative managers can use these maps for identifying high-risk areas and thus to prioritize conservation actions to those areas. Further, this information is useful to them for future surveying and monitoring efforts, and designing conservation strategies and management plans.

## Supplementary Information

Supplementary Information 1.

Supplementary Information 2.

## Data Availability

Data from the current study are available from the corresponding author upon reasonable request.

## Code availability

R script is available from the corresponding author.
