# Supplementary material for: Global potential distribution prediction of Xanthium italicum based on Maxent model
Source: Sci Rep. 2021 Aug 16;11:16545. doi: 10.1038/s41598-021-96041-z (PMC8368065; doi:10.1038/s41598-021-96041-z)
Supplement: Supplementary file 2 — Supplementary Information 2. [file 41598_2021_96041_MOESM2_ESM.docx]

| Measure | Value | Reference | Interpretation |
| --- | --- | --- | --- |
| Kappa (K) | 0.765 | Landis and Koch 1977 | Good agreement |
| Normalized Mutual  Information (NMI) n(s) | 0.830 | Fielding and Bell 1997 | Good prediction |
| True Skill Statistic (TSS) | 0.934 | Allouche et al. 2006 | Performance is better than the random model |

Table S2 Confusion matrix-derived measures of classification accuracy for the MaxEnt model output map of *X. italicum*
